# Disease Resistance and the Definition of Genetic Enhancement

**DOI:** 10.3389/fgene.2017.00040

**Published:** 2017-04-10

**Authors:** Derek So, Erika Kleiderman, Seydina B. Touré, Yann Joly

**Affiliations:** Centre of Genomics and Policy, Department of Human Genetics, McGill University, MontrealQC, Canada

**Keywords:** enhancement, gene editing, gene therapy, CRISPR/Cas9, CCR5, HIV, disease resistance

## Abstract

Recent gene editing experiments carried out in human embryos have raised the question of whether interventions like the introduction of a CCR5-Δ32 deletion, which could provide heritable resistance to HIV infection, ought to be considered enhancements. Many authors have used the term “enhancement” in different ways, some based on patients’ biomedical outcomes and others on their social context. These classifications are often considered overly imprecise. Nevertheless, the concept of “enhancement” could affect the ways in which these applications are regulated in different jurisdictions, the availability of coverage by insurers or public health care, and the force of public opinion in shaping future policy on gene editing. In order to ethically situate resistance to communicable disease with reference to other techniques, this article provides an overview of its similarities and differences with disease gene therapy in embryos, gene therapy in consenting adults, and vaccination. In discussing key ethical features of CCR5-Δ32 deletion (including its frequency in various populations, biological mechanism, benefits for individuals, and use in previous clinical trials) we offer some potential guideposts for the continuing discussion on how to classify “enhancements” in the age of CRISPR gene editing.

## Introduction

Recent scientific advances have heightened the debate over using “gene-editing” technologies like the CRISPR/Cas9 system (Clustered Regularly Interspaced Short Palindromic Repeats; CRISPR-associated protein 9) to make heritable modifications to the human genome. These ongoing international discussions were partly catalyzed by two proof-of-principle experiments performed in China using non-viable human embryos. The first study, published in 2015, attempted to modify the *HBB* gene, which is involved in the genetic blood disorder beta-thalassemia (Liang et al., 2015). The following year, a second Chinese team published the results of a study which, rather than targeting a genetic disease locus, attempted to introduce the *CCR5-Δ32* gene variant, a 32-bp deletion that prevents some strains of HIV from entering white blood cells via the CCR5 receptor protein (Kang et al., 2016).

These two experiments have raised the novel question of whether gene editing aimed at providing resistance to communicable diseases (RCD) ought to be considered similar to therapeutic editing from an ethical perspective, or whether it ought to be classified as a form of “enhancement.” In this article, we examine the reasons why this distinction might be important to the uptake of gene editing, and provide examples of biotechnologies that have raised similar ethical concerns. We also discuss the merits and risks of describing traits like HIV resistance as enhancement at this stage in the development of governance for CRISPR.

## Why is the Classification of “Enhancement” Significant?

For many years, bioethicists have written about the use of genetic engineering to “enhance” human traits, including its consequences for distributive justice, discriminatory social norms, and the preservation of children’s autonomy (Parens, 1998). While speculative modifications to intelligence, strength, or attractiveness are more frequently discussed than *CCR5-Δ32* editing, it is possible that they raise similar moral questions and deserve to be classified in the same way. Although the question of different labels for gene editing can seem overly abstract, the loosely defined category of “enhancement” could affect future uses of gene editing technologies through its use in regulation, health policy, and public discourse.

### Regulation

Over 40 jurisdictions have written regulations on human germline genetic modification, most of which ban the practice in some form (Araki and Ishii, 2014; Isasi and Knoppers, 2015). For instance, Australia, Canada, France, and Germany have strict laws against altering the human germline. While similarly restrictive approaches have been adopted by countries such as China, India, and Japan, the attendant sanctions are often unclear and may not be legally enforceable (Araki and Ishii, 2014; Isasi et al., 2016). The lack of guidance and oversight in these countries could weaken public trust in science regulation (Caplan et al., 2015).

Many of these policies reflect policymakers’ fears of dystopian and disruptive use of technologies such as human cloning (Knowles and Kaebnick, 2007; Knoppers et al., 2017). Their scope is frequently outlined in abstract or subjective language (Isasi et al., 2016): the UN *Declaration on Human Cloning* instructs member states to prohibit techniques “that may be contrary to human dignity” (United Nations, 2005); pan-European regulations on clinical trials prohibit “modifications to the subject’s germ line genetic identity”; Israeli law allows genetic interventions only where “human dignity will not be prejudiced” (ISRAEL, 1999; European Parliament, 2014). Regulations from Germany and India also prohibit germline enhancement and express concern about eugenics (Indian Council of Medical Research, 2000; Interdisciplinary Study Group “Gene Technology Report”, 2008). Thus, classifying RCD as an enhancement could result in it being more strictly regulated or proscribed in some jurisdictions.

The label of enhancement could also prevent RCD from falling under exemptions in some laws which prohibit germline modification generally but permit interventions for therapeutic purposes (Isasi et al., 2016). Treatment and enhancement are often defined in opposition to one another in the context of genetic modification (Committee on Human Gene Editing, and National Academies of Sciences, Engineering, and Medicine, 2017). Thus, a preventive “treatment” for HIV might be included in these exemptions, while an “enhancement” might receive stricter scrutiny. As a related example, the Council of Europe’s (1997) *Oviedo Convention* states that genomic modification “may only be undertaken for preventive, diagnostic or therapeutic purposes and only if its aim is not to introduce any modification in the genome of any descendants.” It is possible that, in some countries, “correcting” a genetic disorder would not count as the introduction of a heritable modification (Ishii, 2015). However, it seems likely that the introduction of an “enhancement” would remain more strictly regulated in these cases.

### Health Coverage

Even if gene editing to provide RCD in human embryos is eventually permitted in some jurisdictions, access to such interventions may be restricted by insurers or public health care systems unwilling to subsidize costly “enhancements” (Buchanan et al., 2000). Glybera, the first gene therapy approved in Europe, was introduced at a cost of €1.1 million per patient, making it the world’s most expensive medicine and resulting in disputes over insurance reimbursement. The second, Strimvelis, cost €594,000 (Abou-El-Enein et al., 2016). Although RCD for embryos would not necessarily be as expensive, it would have to be performed alongside one or more cycles of IVF (*in vitro* fertilization), incurring further medical, economic, and social costs. Although the ethical ramifications of relying on IVF for gene editing are still poorly understood, it is beyond the scope of this article to outline these issues here (Zimmerman, 1991; Chambers et al., 2013; Werner-Felmayer and Shalev, 2015).

In the same way that cosmetic surgeries are generally excluded from both private insurance policies and public programs like the United States’ Medicare and Medicaid, both types of payer might choose to classify ambiguous cases as enhancements in order to justify considering them as elective rather than medically necessary procedures. This could allow them to avoid paying for expensive new technologies which are also likely to be socially controversial (Mehlman, 1999). However, some authors suggest that therapeutic gene editing could help reduce overall health care expenditures as well as the associated costs of caring for people with cystic fibrosis, sickle cell anemia, and other genetic diseases (Zimmerman, 1991; Walters and Palmer, 1997; Resnik et al., 1999). Members of the biotechnology industry may also advocate labeling gene editing as treatment, given their commercial interests in the widespread use of CRISPR and related technologies.

### Public Opinion

The development and use of new biotechnologies can be affected by public attitudes, which influence resource allocation, “political policy,” and participation rates in experimental clinical studies (McCaughey et al., 2016). It is widely agreed that public consultation is an important step in the present ethical deliberation over the appropriate uses of CRISPR/Cas9 in humans. For instance, the American College of Medical Genetics’ Board of Directors have urged “broad public debate” to inform this decision (ACMG Board of Directors, 2017), while the organizers of the International Summit on Human Gene Editing stated that clinical germline editing would require “broad societal consensus about the appropriateness of the proposed application” (Baltimore et al., 2016).

However, societal views are difficult to assess. More public surveys on gene editing have been carried out in the United States than any other country, yet there is still not enough data to indicate a clear trend. A large number of respondents, although not a majority, generally accept the prevention of inherited genetic diseases. Most respondents draw a much stronger line at modifications aimed at improving or “enhancing” physical or psychological traits (Blendon et al., 2016; Funk et al., 2016). Despite this clear discrepancy, no survey has ever asked a question specific enough to elicit opinions on providing future children with RCD.

This situation has limited experts’ ability to make evidence-based theories regarding public opinion on gene editing, as well as policymakers’ desire to take societal values into account. It also raises doubts whether most laypeople have sufficient knowledge of genetics to provide an informed opinion at this time, although these beliefs could solidify as the technology becomes more prominent. Labeling ambiguous interventions like *CCR5* editing as “enhancements” could reduce support from the general public, regardless of the validity of these concerns; these opinions may carry significant consequences for policy development.

## Can Resistance to Communicable Diseases be Classified as Human “Enhancement”?

Despite these potential effects, the term “enhancement” is notoriously blurry. Definitions may refer to the procedure’s means or its intended outcome. They can also focus on broad social and philosophical issues, or on specific impacts upon individual patients (Committee on Human Gene Editing, and National Academies of Sciences, Engineering, and Medicine, 2017). In the former framework, authors frequently question whether gene editing would represent a primarily competitive advantage, or an “absolute good” benefiting its recipients independent of their social context (Buchanan et al., 2000; Sandel, 2004; Fox, 2007; Cohen, 2014; Elhauge, 2014; Committee on Human Gene Editing, and National Academies of Sciences, Engineering, and Medicine, 2017).

In the latter, more individual approaches, health is often considered to follow a continuum with disease on the bottom, “enhanced function” on top, and health falling in the middle (Buchanan et al., 2000). Some consider any intervention which moves someone further up the spectrum to be an enhancement, regardless of the starting point or the endpoint (Walters and Palmer, 1997; Quigley and Harris, 2009). Other authors define enhancement as any change that raises someone into the “better than well” range (Greely, 2008; de Melo-Martín, 2010). However, RCD editing as typically envisioned would prevent a healthy person from potentially falling lower on the spectrum, meaning neither definition would apply.

Parens (1998) suggests simply adding the category of “prevention,” but this does not tell us whether RCD would be treated as an enhancement by the actors discussed above unless enhancement, prevention and treatment are mutually exclusive. This assumption may not be useful from a regulatory, normative, or scientific perspective. First, many authors have referred to identical interventions using each of the three terms. Second, reference points on the health continuum depend both on the population and the course of medical progress. Third, genetic interventions could involve very similar methods and outcomes, meaning that treatments intended for disease and enhancements intended for healthy patients might be equivalent from a purely biomedical perspective. And fourth, these categories may not capture relevant social attitudes or realistic policy options (Walters and Palmer, 1997; Mehlman and Botkin, 1998; Elhauge, 2014; Committee on Human Gene Editing, and National Academies of Sciences, Engineering, and Medicine, 2017). Given these difficulties, it may be more helpful to examine RCD’s similarities and differences with interventions about which we are relatively secure in our moral intuitions, including gene therapy in embryos, gene therapy in adults, and vaccination.

### Gene Therapy in Embryos

At first glance, the two studies editing *HBB* and *CCR5* in non-viable human embryos seem very similar: the only significant difference in their methods was the design of different guide RNAs for targeting purposes (Liang et al., 2015; Kang et al., 2016). According to the continuum-based definitions cited above, correcting thalassemia would seem to fall squarely within the purview of medicine. Norman Daniels (1985) argues that the only obligatory forms of care are those which restore “species-typical functioning” on a biological level in order to give patients a “normal range of opportunity” in society. While definitions of medical “normalcy” have been widely debated (Committee on Human Gene Editing, and National Academies of Sciences, Engineering, and Medicine, 2017), that question is beyond the scope of this article, and we believe most people would agree that severe genetic disorders do not represent typical function and result in a restricted range of opportunities compared to “healthy” people. A similar argument could theoretically be made for *CCR5* editing and the limitations on opportunity imposed by HIV/AIDS. In this case, the absence of HIV infection might be considered “normal” or “species-typical.”

One objection to this interpretation might be that wild type, HIV-vulnerable *CCR5* alleles should represent normal functioning, since they represent the large majority of people in every ethnic group. In Northern Europe, only up to 14% of the population may have copies of the *CCR5-Δ32* allele, while in East Asian populations, the HIV-resistant population is functionally nil (Stephens et al., 1998). In fact, it has previously been suggested that introducing natural variants of sufficient rarity into an embryo should be considered enhancement. Yet as with the concept of “normalcy,” the question of where to draw the line for rarity in a biological population remains somewhat open, and allele frequency itself can change over time or geography (Walters and Palmer, 1997; Parens, 1998; Committee on Human Gene Editing, and National Academies of Sciences, Engineering, and Medicine, 2017).

RCD could also be compared with interventions which, rather than targeting clear-cut disorders like beta-thalassemia, attempt to reduce genetic predispositions to adult-onset diseases. Just as human behavior interacts with genotype to influence cancer and diabetes risks, *CCR5* editing would also modulate risks dependent on environmental exposure. As such, RCD may represent an enhancement in that it would allow a future person to live with fewer worries or greater freedom than their peers. While the use of preimplantation genetic diagnosis to avoid severe genetic disorders has many proponents, the selection of embryos based on Alzheimer’s risk has been widely criticized by ethicists as an overreach of parental decision-making (Robertson, 2003; Anderson et al., 2015). If there is an ethical boundary between limiting future risks and addressing conditions with well-defined existing etiology, it might be prudent to classify the former as enhancement.

### Gene Therapy in Adults

One appeal of the comparison between embryo editing for RCD and gene therapy in adults is that both methods may involve the same genetic “edit.” Indeed, somatic *CCR5-Δ32* editing in T cells has already been tested as a treatment for HIV-positive adults (Tebas et al., 2014). These methods are considered ethically acceptable provided they satisfy requirements regarding risk–benefit ratio and informed consent (Lander, 2015; Rodriguez, 2016). However, germline modification raises additional concerns about unpredictable, inherited effects on future generations who would have no say in the decision (Rodriguez, 2016).

It is not clear that consent is relevant to the classification of enhancement. Many theorists differentiate acceptable from unacceptable interventions based on whether they maximize the “open future” of children, providing them with the means to achieve their own projects, or whether they restrict children to lives following their parents’ value systems (Feinberg, 1980; Habermas, 2003; Agar, 2004). Yet even philosophers with vastly different views on human gene editing agree that it could prevent many sorts of goals from being sidetracked by illness (Buchanan et al., 2000; Habermas, 2003; Quigley and Harris, 2009). RCD is unlikely to represent the threat to identity or authenticity feared by some of the legislators discussed above.

The second relevant difference lies in these methods’ effect on future generations. Assuming people have genuine interests in the health of their immediate descendants, it might be argued that RCD editing represents an enhancement compared to somatic therapy. This possibility, combined with the high price of gene editing, evokes longstanding fears about societal stratification, discrimination against the “genetic underclasses,” and political instability (Walters and Palmer, 1997; Parens, 1998; Agar, 2004; Joly et al., 2013). However, broadly subsidized RCD could be seen as a public health measure. Similar to the way in which vaccination creates “herd immunity,” reducing the total number of people vulnerable to communicable diseases could help shield those without the protective allele. For instance, South Africa’s representative to the International Summit on Gene Editing discussed *CCR5* gene therapy as a potential strategy in dealing with the public health burden of HIV/AIDS in Africa (Moodley, 2015).

### Vaccination

Like embryonic *CCR5* editing, vaccination often involves manipulating someone’s immune system without their consent in order to boost their resistance to infections. Interestingly, the question of whether vaccines represent enhancement has already been discussed in the literature (Bostrom and Roache, 2007; Committee on Human Gene Editing, and National Academies of Sciences, Engineering, and Medicine, 2017). Since vaccination is morally accepted by most stakeholders, those who reject enhancement have had to find ways to exclude vaccination from its definition (Douglas, 2013). Daniels (2000), for instance, states that vaccinations “exploit more fully our immune capabilities rather than extending them.” However, many ethicists describe vaccination as a clear enhancement beyond species-typical functioning (Walters and Palmer, 1997; Harris, 2007; Quigley and Harris, 2009; Roberts, 2014), and those who support more permissive uses of human gene editing often cite it as proof that enhancement is already being widely practiced (Parens, 1998).

In response, it could be argued that RCD in the form of *CCR5-Δ32* editing does not actually represent a functional upgrade to immune activity the way vaccination does. It merely changes the structure of the CCR5 receptor in a way that limits HIV entry into host cells (Lopalco, 2010). Furthermore, this allele appears to be associated with a significant increase in susceptibility to West Nile virus (Glass et al., 2006; Moodley, 2015). On second glance, even a successful *CCR5-Δ32* deletion might be viewed not as an objective enhancement so much as a deliberate trade-off, with both advantages and disadvantages depending on the medical context (Lander, 2015; Gyngell et al., 2016).

## Conclusion

Recent experiments involving human embryos have raised ethical and legal questions about the editing of genes like *CCR5* in order to promote disease resistance. Given the longstanding bioethical debate over human “enhancement,” the labeling of these techniques could have significant effects on their eventual clinical uses. First, regulations in many jurisdictions refer to subjective concepts which could be used to exclude enhancements. Second, both insurance companies and public health care systems could make or interpret policy in order to avoid paying for such interventions. Third, ethics deliberation and political decision-making could be influenced by public fear—whether rational or irrational—of dystopian futures following from genetic enhancement.

Although the concept of enhancement is nebulous, confusing, “freighted with erroneous assumptions and ripe for abuse” (Parens, 1998), it seems too entrenched in our language to be ignored or replaced. While actual consensus about its definition would represent an important breakthrough (Hotze et al., 2011), we are not suggesting a new definition in this article. Rather, our investigation of RCD has demonstrated a number of ways in which using the ambiguous label of “enhancement” as a guiding principle can be limiting for the bioethical debate. Arguments for or against new interventions should appeal to more concrete ethical concerns, such as the provision of competitive advantages against other members of society. Regulators should also consider using more specific language in governance documents. In the present context, however, we suggest that ambiguous cases be more pragmatically classified as enhancement or non-enhancement based on considerations of the public good. While germline gene editing does not seem efficient as a public health measure, it also does not appear to raise significant ethical issues beyond the other techniques discussed above. Therefore, we do not see a strong case for considering it an enhancement in the present context.

For the purposes of this article’s more philosophical arguments, we have assumed the eventual safety and efficacy of embryonic gene editing. However, the technology is currently agreed to be unsafe for clinical use (Liang et al., 2015; Baltimore et al., 2016; Kang et al., 2016; Committee on Human Gene Editing, and National Academies of Sciences, Engineering, and Medicine, 2017). Given our lack of experience with these technologies, the use of CRISPR in a human embryo at this stage would be more likely to produce mosaicism and off-target effects than the desired enhancement. Modifications capable of being inherited by future generations must also be held to especially rigorous safety standards. The risk of introducing disorders into the germline of a healthy embryo, or of providing RCD to some diseases at the cost of increased vulnerability to others, ought to be taken into account in the calculus of labeling interventions as enhancements.

It should also be noted that many ethicists argue against editing human embryos regardless of whether it represents enhancement. They express concern that any intervention represents a slippery slope toward more problematic forms of gene editing (Annas et al., 2002). Further dialog on this topic can help us avoid inadvertently facilitating morally blurry interventions. We should endeavor to predict conflicts which could arise from different perceptions of these technologies, while continuing to examine the relation between our ethical and regulatory frameworks and stakeholders’ views on the concept of enhancement.

## Author Contributions

DS conceived the article subject, wrote one-third of the first draft, and performed final edits on each draft. EK wrote one-third of the first draft and edited the manuscript. ST wrote one-third of the first draft and edited the manuscript. YJ oversaw the writing and edited the manuscript.

## Conflict of Interest Statement

The authors declare that the research was conducted in the absence of any commercial or financial relationships that could be construed as a potential conflict of interest.
